# Horizontal sliding of kilometre-scale hot spring area during the 2016 Kumamoto earthquake

**DOI:** 10.1038/srep42947

**Published:** 2017-02-20

**Authors:** Takeshi Tsuji, Jun’ichiro Ishibashi, Kazuya Ishitsuka, Ryuichi Kamata

**Affiliations:** 1 International Institute for Carbon-Neutral Energy Research (I2CNER), Kyushu University 744 Motooka, Nishi-ku, Fukuoka 819-0395, Japan; 2 Department of Earth Resources Engineering, Kyushu University 744 Motooka, Nishi-ku, Fukuoka 819-0395, Japan; 3 Faculty of Science, Kyushu University 744 Motooka, Nishi-ku, Fukuoka 819-0395, Japan; 4 Fukada Geological Institute 2-13-12 Komagome, Bunkyo-ku, Tokyo 113-0021, Japan; 5 Chiiki-Shigen-Kaihatsu 2-5-15 Tatsuta Jinnai, Kita-ku, Kumamoto 861-8005, Japan

## Abstract

We report horizontal sliding of the kilometre-scale geologic block under the Aso hot springs (Uchinomaki area) caused by vibrations from the 2016 Kumamoto earthquake (Mw 7.0). Direct borehole observations demonstrate the sliding along the horizontal geological formation at ~50 m depth, which is where the shallowest hydrothermal reservoir developed. Owing to >1 m northwest movement of the geologic block, as shown by differential interferometric synthetic aperture radar (DInSAR), extensional open fissures were generated at the southeastern edge of the horizontal sliding block, and compressional deformation and spontaneous fluid emission from wells were observed at the northwestern edge of the block. The temporal and spatial variation of the hot spring supply during the earthquake can be explained by the horizontal sliding and borehole failures. Because there was no strain accumulation around the hot spring area prior to the earthquake and gravitational instability could be ignored, the horizontal sliding along the low-frictional formation was likely caused by seismic forces from the remote earthquake. The insights derived from our field-scale observations may assist further research into geologic block sliding in horizontal geological formations.

In the 2016 Kumamoto earthquake, a series of temblors occurred along the Hinagu-Futagawa fault system in the central area of Kyushu Island, Japan (Fig. 1a)^1^,^2^,^3^,^4^. The series began along the Hinagu fault system (oriented NNE–SSW) with a Mw 6.2 foreshock on 14 April. The largest event of the series (Mw 7.0) occurred 28 hours later (16 April) along the Futagawa fault system (oriented NE–SW), presenting a right lateral and normal motion^1^,^3^. Surface deformation caused by ruptures along these faults was clearly observed; the tectonic motion destroyed many houses and a considerable amount of infrastructure^5^. Intensive open fissures formed in the Aso hot spring area (Uchinomaki area) northwest (NW) of the Mount Aso caldera (Fig. 2c). The fissures have been often interpreted as surface traces of earthquake-induced faults. However, using differential interferometric synthetic aperture radar (DInSAR) we identified anomalous deformation around the Aso hot spring area that cannot be explained by previously identified fault ruptures. Certain hot springs in this area became dormant after the 2016 earthquake (green dots in Fig. 2c), whereas others experienced increased flow compared with their pre-earthquake state (red dots in Fig. 2c).

In this study, we report the mechanisms underlying the anomalous deformation and the variations of the hot spring supply using DInSAR data, field observations, descriptions of the temporal variations of the hot springs, and data from a borehole camera. The borehole observations demonstrate directly that the kilometre-scale hot spring area moved along the horizontal formation at a depth of ~50 m.

## Results

**Figure 1:**
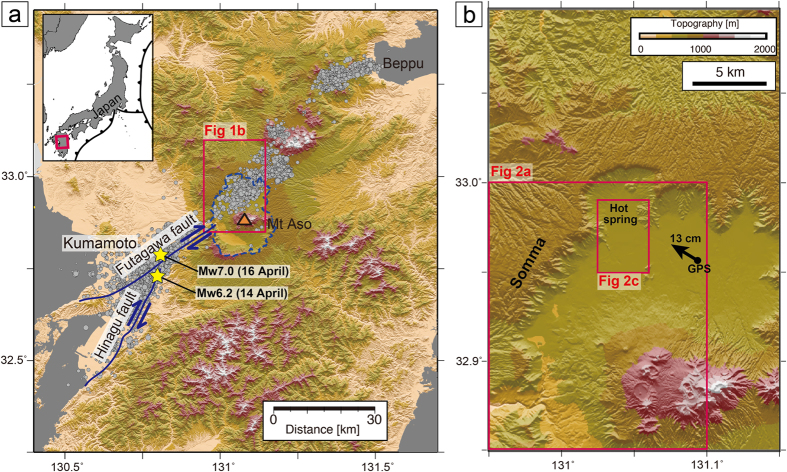
Map of central Kyushu Island, Japan. (**a**) Locations of the faults (blue lines) and hypocentres of large earthquakes (yellow stars) and aftershocks (grey dots) in the 2016 Kumamoto earthquake. These faults were recognized as active faults before the 2016 earthquake. Dashed outlines show the Aso caldera. (**b**) Enlarged map around the Aso hot spring (Uchinomaki) area. Black arrow indicates the direction of surface deformation during the earthquake (15–16 April) based on GPS data (GEONET station)^18^. We used the 10-m mesh digital elevation model (DEM) published by the Geospatial Information Authority of Japan (http://www.gsi.go.jp/). These figures were drawn using the Generic Mapping Tools^24^.

**Figure 2:**
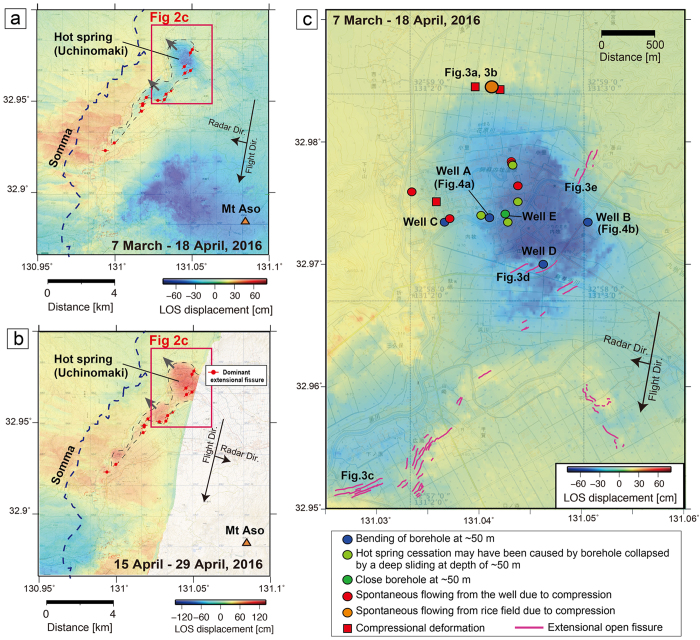
Surface deformation and variation of hot spring supply. (**a**) DInSAR-derived surface deformation acquired from 7 March to 18 April 2016. The area is shown as a red rectangle in Fig. 1b. The radar beam direction is shown by a black arrow. The colour scale shows the surface deformation in the Line-Of-Sight (LOS) direction. (**b**) DInSAR-derived deformation acquired from 15 to 29 April 2016. The radar direction of this result (in a left-looking manner during descending orbits) is different from that shown in panel (**a**). (**c**) Surface deformation around the Aso Hot Spring (Uchinomaki) area. Dots indicate the wells where temporal variations of the hot springs were observed. Thin red lines represent open fissures revealed by field observations and interpretations of satellite data. We used the regional map published by the Geospatial Information Authority of Japan (http://www.gsi.go.jp/).

To reveal the surface deformation and mechanisms underlying the temporal and spatial variations of the hot spring supply, we first applied DInSAR analyses^6^,^7^,^8^,^9^,^10^. Two SAR datasets acquired before and after the mainshock on 16 April, 2016 were compared. One dataset included the interferometric pair recorded from 7 March–18 April 2016, acquired in a right-looking manner during descending orbits (PATH:23/FRAME:2950) (Fig. 2a). The other dataset included the pair recorded from 15–29 April 2016 (PATH:28/FRAME:2930), acquired in a left-looking manner during descending orbits (Fig. 2b). The results of the DInSAR analysis showed local deformation along the NW edge of the Mount Aso caldera from the Aso hot spring (Uchinomaki) area to its southwestern (SW) area. The area encompasses a flat plain surrounded by hills (i.e., Somma of Mount Aso) (Fig. 1b, Supplementary Fig. S1). The area was directed away from the satellite in the right-looking image (black arrows in Fig. 2a) and approached the satellite in the left-looking image (black arrows in Fig. 2b), indicating surface displacement consistent with westward motion. The local movement derived from DInSAR cannot be explained by the main fault rupture, which has a right lateral motion. By considering two surface deformations from different radar directions, the maximum westward movement of the hot spring area is ~1 m (Fig. 2c). Because the movement of the region may not be purely in the east–west direction (i.e., the radar direction), the maximum displacement may be larger than 1 m. The shape of the geologic block that moved in the Aso hot spring (Uchinomaki) area is nearly circular with a diameter of ~2 km (Fig. 2c). The deformation within the block that moved (i.e., the central part of the hot spring area) is not clearly visible from the DInSAR results (Fig. 2c) or field observations, suggesting that the geologic block moved smoothly during the mainshock.

**Figure 3:**
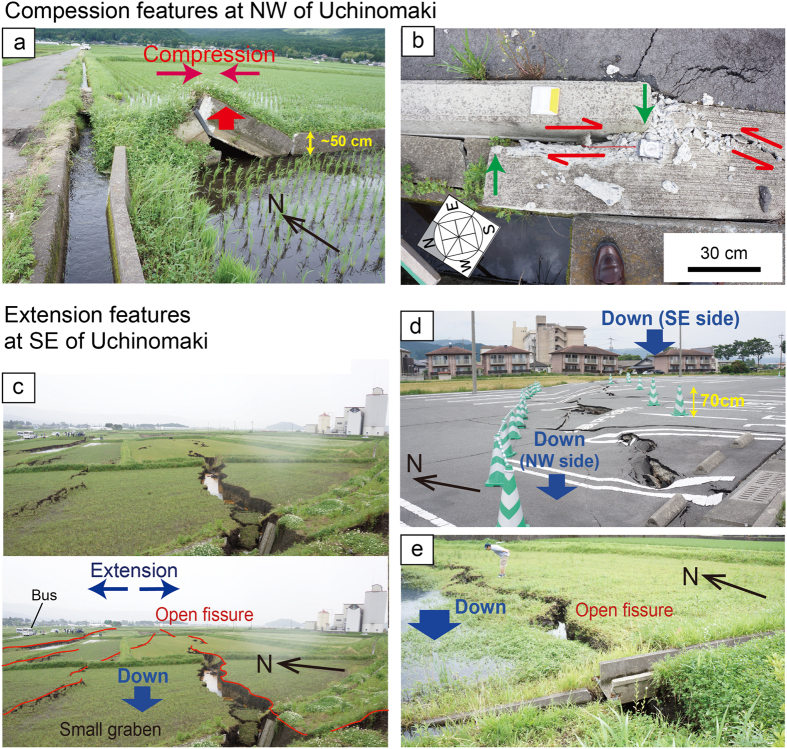
Pictures of surface deformation around the shifted geologic block. (**a,b**) Compressional deformation and (**c–e**) extensional deformation; see locations in Fig. 2c. In panel (**a**), the bent concrete was caused by compression. In panel (**b**), the concrete edges marked by the green arrows were originally attached but were shortened by compaction. (**c**) Small graben (pull-apart basin) generated by the extension forces. Red lines show the locations of the open fissures.

However, intensive deformation, which is marked by open fissures, occurred at the edge of the sliding geologic block. Our field observations in these areas documented numerous open fissures approximately 1 m wide along the SE edge of the block that moved (red lines in Figs 2c and 3c–e). Small pull-apart basins were generated by a combination of extensional fissures (Fig. 3c). Lateral offset did not appear to occur along these fissures. Figure 3d shows the subsidence on the NW side of the fissure (blue arrow) at the front of the picture, and the subsidence on the SE side of the fissure at the back of picture; these features represent extension. Thus, these intensive fissures were generated by extension caused by >1 m of horizontal movement of the geologic block.

Compressional features (red rectangle in Fig. 2c), caused by the collision of the moving block, were observed along the NW edge of the ~2 km geologic block (Fig. 3a,b). For example, the collapse of the concrete shown in Fig. 3b demonstrates a compression of ~60 cm in the horizontal direction at this location. The surface deformation at the compressional region appears less intensive than the extensional deformation. We could not find clear compressional features in the rice field which consists of soft sediment. The soft sediment may absorb the compressional strain, and compressional features in soft sediment are not as prominent as extensional ones for similar deformation. However, the concrete sidewalk, which is much stiffer than the sediment, showed close to 1 m of compressional deformation (Fig. 3a,b). In this compression area, new seepages from fissures a few centimetres in width were widely distributed (around orange dot in Fig. 2c), and spontaneous fluid flow from the wells occurred (red dots in Fig. 2c) after the earthquake. This spontaneous flow may have been caused by an increase in pore pressure related to the compaction associated with the block movement.

**Figure 4:**
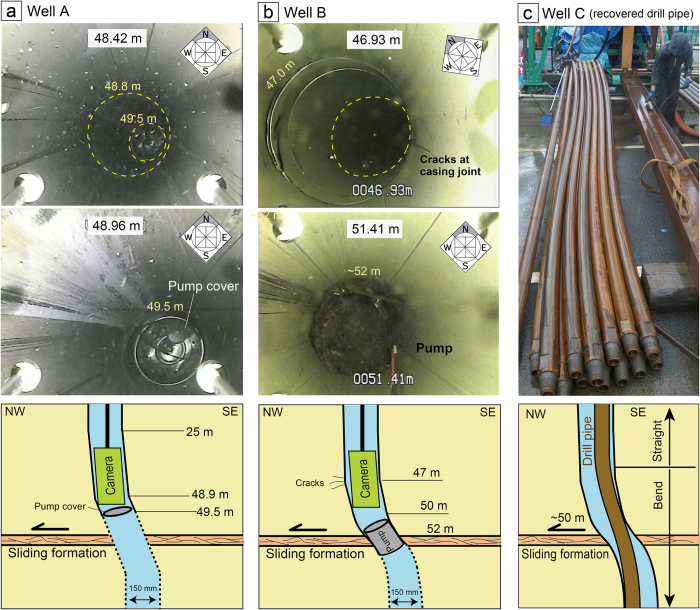
Pictures of the boreholes and drill pipes that deformed during the mainshock on 16 April, 2016. (**a,b**) Images taken inside the casing pipe show the bending of the borehole at ~50 m. The diameter of the borehole casing is 150 mm. The yellow dashed lines in panels (**a** and **b**) indicate the same depth within the casing. Lower panels show a schematic image of the observed borehole. (**c**) The drill pipe installed within well C was bent during the 16 April earthquake; un-deformed drilling pipes (left) and the deformed pipes (right). Each pipe is 6 m long and 88.9 mm in diameter. The locations of the wells are displayed in Fig. 2c.

However, while new emissions of fluid were observed in the NW parts of the study area, the flow of several hot springs located in the central part of the sliding block ceased after the mainshock (green dots in Fig. 2c). To reveal the mechanisms underlying this hot spring flow cessation, we used a borehole camera to characterize the dynamic deformation of the subsurface hydrothermal reservoir. The subsurface observations demonstrate that the borehole casings collapsed or bent at a depth of ~50 m during the mainshock on 16 April (Fig. 4). The cameras in wells A and B (Fig. 2c) were tilted in the NW direction at ~50 m depth (Fig. 4; Supplementary Video S1). The boreholes, which were originally vertical, bent at ~50 m because of the NW movement of the shallow formation. The casing pipe is made of stainless steel; therefore, although the sliding along the detachment (~1 m) was larger than the casing diameter (150 mm), the strength of the stainless steel casing ensured that the borehole remained open. Indeed, the casing pipe at well A was gently tilted below ~25 m and largely deformed below 49 m (see Fig. 4a). At well B (Fig. 4b), the borehole was largely tilted at depths below 47 m. If the average tilt angle of the borehole from 47 to 52 m is 5° and symmetrical borehole deformation across the sliding formation is assumed, the ~1 m horizontal sliding at 52 m depth can be explained (Fig. 4b). Because wells A and B are located at the edge of the horizontal shifted block, as inferred from DInSAR (Fig. 2), the amount of horizontal movement at these wells is smaller than in the central part of the shifted block. Indeed, many wells where the hot spring flow ceased are located in the central part of the shifted block (green dots in Fig. 2c); these wells may have completely collapsed because of the large displacement (>1 m horizontal sliding) at the detachment surface at ~50 m.

The pumps that supply the hot spring water at wells A and B (Fig. 4a,b) were originally deployed at a depth of several hundred metres; however, when we tried to recover the pumps after the earthquake, they were stacked at ~50 m. This indicates significant deformation at the horizontal formation at ~50 m. At well D (Fig. 2c), the pump could not be recovered and was stacked at ~60 m because of borehole bending, suggesting that the deformation occurred at a depth shallower than 60 m (and maybe deeper than 50 m). The drill pipe installed in well C (Fig. 2c) was bent below 50 m during the mainshock on 16 April (see Fig. 4c). The drilling at well C was operating from March 2016 (before the mainshock). The drill pipe was functioning effectively in the borehole after the 14 April earthquake. Thus, the drill pipe was bent during the 16 April mainshock. When we tried to recover the drill pipe through the bent section of the borehole (at ~50 m), all the drill pipes deeper than 50 m were bent as shown in Fig. 4c. This clearly demonstrates that the borehole bending was caused by the mainshock. At well E where the hot spring supply was halted after the earthquake (dark green dots in Fig. 2c), the logging tool could not penetrate deeper than 50 m, suggesting that the well had collapsed at ~50 m. Many wells where the hot-spring flow stopped (green dots in Fig. 2c) are located in the most displaced area (i.e., the central part of the hot spring area). When new wells were drilled through the ~50 m sliding horizon close to the wells where the hot spring flow had stopped after the earthquake, the hot spring supply was recovered, suggesting that these boreholes collapsed at ~50 m due to horizontal sliding in the 2016 Kumamoto earthquake. The hydrothermal activity (or the source of the hot springs) deeper than 50 m was not considerably influenced by the 2016 earthquake.

**Figure 5:**
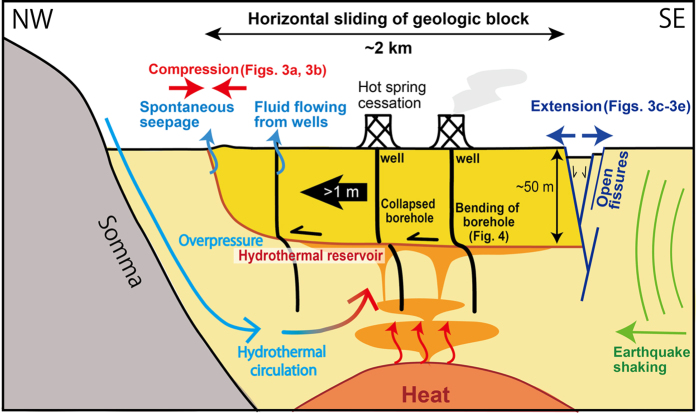
Schematic image of the horizontal sliding of the Aso hot spring area. The earthquake motion caused the kilometre-scale geologic block to slide NW along the horizontal detachment surface at ~50 m depth.

All the observed data suggest that the shallow geologic block of the Aso hot spring slid over 1 m in the NW-direction along a specific geologic layer at ~50 m (Fig. 5). The deformed horizon at 50 m depth appears to act as a basal detachment fault. Residents in the hot spring area were aware that boreholes had collapsed in prior earthquakes [personal communication with residents in Uchinomaki, 2016], indicating that the shallow geologic block had been displaced in the past.

## Discussion

Landslides (or lateral spread) and horizontal slides in shorelines or river embankments have frequently been reported^11^,^12^,^13^,^14^. These movements are generally induced by gravitational instability. However, the slope inclination for the sliding direction (NW) in the Aso hot spring area is less than 0.06° (Supplementary Fig. S2), which is much less than the reported minimum slope inclination for lateral spreads^12^,^15^. Furthermore, the depth of the sliding detachment (~50 m) varies very little in the Aso hot spring area (Fig. 2c). For example, the depth of the sliding formation at wells A and B, which are ~1 km apart, is similar. Several logging datasets (Supplementary Fig. S3) further support similar lithology distributions at ~50 m depth. This suggests that the horizontal sliding was not caused by gravitational forces, although gravitational instability cannot be ruled out completely. Horizontal sliding of a kilometre-scale geological block without gravitational forces has not been reported. Furthermore, because the geologic block appears to have moved without strain accumulation, the displacement could only have been triggered by the shaking of the remote earthquake. The Aso hot spring area is >5 km from the closest fault plane^1^,^2^,^3^,^4^. This region is located at the NE edge of the Futagawa fault which has right-lateral motion (Fig. 1a); thus, the earthquake shaking was relatively large (517 gal in the Uchinomaki area)^16^. Although the NW movement of the Aso hot spring cannot be clearly explained by the right-lateral motion of the Futagawa fault (NE–SW strike), the location of the Aso hot spring (NE edge of the Futagawa fault) and the complexity of the lithology at the volcanic caldera may be related to the NW movement. Indeed, geomorphic image analysis^17^ demonstrated that a large area of the NW Aso Caldera moved N to NW. The GPS data close to the Aso hot spring^18^ also showed NW horizontal movement (black arrow in Fig. 1b).

Shallow geologic block movements (e.g., landslides) are also enhanced by soil liquefaction or increased pore pressure along the sliding formation^11^,^12^. The pore pressure in the hot spring area was originally higher than the hydrostatic state because of fluid flowing from certain wells. The formation at ~50 m where the horizontal slip occurred is a layered structure consisting of silt and conglomerate (gravel), and is identified as a low natural gamma-ray layer in the logging data (Supplementary Fig. S3). The conglomerate layer at 50–70 m is the hydrothermal reservoir of the hot spring with higher pore pressure. During the earthquake, the pore pressure at the porous conglomerate layer may have increased by the crushing of the gravel along the sliding surface^11^ and by the sealing of the fluid beneath the low-permeability silt layer. Therefore, the hydrothermal reservoir formation (or conglomerate layer) can be considered a detachment fault (Fig. 5). In particular, the sequence of earthquakes, including the first earthquake on 14 April, may have instantaneously weakened the formation^19^ and slightly increased the pore pressure before the mainshock on 16 April, partially contributing to the horizontal sliding.

The results of our study suggest that the movement of the localized geologic block occurred smoothly above the basal detachment formation and was caused by the seismic forces of the 2016 earthquake, as the geological formation in this region could not accumulate any strain before the earthquake. Thus, the frictional coefficient along the horizontal layer at a depth of 50 m was low. Horizontal sliding along low-friction detachment surfaces without strain accumulation can occur in other geologic settings, such as shallow, low-dipping faults. A large tsunami is often generated by a large displacement along a low-frictional detachment fault close to the oceanic trench axis^20^,^21^,^22^. Our field-scale observations demonstrate that displacement along a shallow, low-dipping fault at low friction could easily occur even without previous strain accumulation.

The intensive deformation and hydrodynamics discussed here have important scientific implications and also provide valuable information for the design and maintenance of local infrastructure and continuous management of the hot spring. If the underground pillars of a building is set deeper than the sliding horizon, the building could be significantly damaged. Furthermore, if the local surface deformation revealed in this study is used for rupture characterizations of the main fault^7^, the results could include errors; therefore, the potential for geologic block sliding should be considered, even in a geological setting of a plain.

## Methods

### Differential Interferometric SAR (DInSAR)

Synthetic aperture radar (SAR) measures the reflectivity of ground targets via the transmission and acquisition of microwave radar waves. The waves propagate through the atmospheric layer without a noticeable signal loss and provide all-weather and 24-hr capabilities. The DInSAR analysis estimates the surface displacement in a line-of-sight direction using the phase information of two SAR observations acquired at different times with similar viewing geometries^6^. Because several physical phenomena contribute to the phase difference, DInSAR processing includes a simulation in which the phase contribution is subtracted because of the topography and orbital trajectory.

The ALOS-2 SAR satellite is the successor to ALOS; it was launched in May 2014, and since then has acquired many SAR images suitable for DInSAR analyses. The data used in this study were acquired by the strip-map mode of ALOS-2 at a spatial resolution of approximately 1.43 m and 1.92 m in the slant-range and azimuth directions, respectively. In this study, we applied multi-look processing (i.e., averaging) with 10 × 10 pixels and used a 10-m mesh digital elevation model provided by the Geospatial Information Authority of Japan to simulate the contribution of the topographic phase. A minimum cost-flow algorithm^23^ was applied to the data for phase unwrapping. To minimise the tropospheric contribution, the phases that correlated with the topography were estimated using a least squares method and then subtracted from the unwrapped interferogram.

## Additional Information

**How to cite this article**: Tsuji, T. *et al*. Horizontal sliding of kilometre-scale hot spring area during the 2016 Kumamoto earthquake. *Sci. Rep.* **7**, 42947; doi: 10.1038/srep42947 (2017).

**Publisher's note:** Springer Nature remains neutral with regard to jurisdictional claims in published maps and institutional affiliations.

## Supplementary Material

Supplementary Video S1

Supplementary Figures
