# Supplementary material for: Horizontal sliding of kilometre-scale hot spring area during the 2016 Kumamoto earthquake
Source: Sci Rep. 2017 Feb 20;7:42947. doi: 10.1038/srep42947 (PMC5317158; doi:10.1038/srep42947)
Supplement: Supplementary Figures [file srep42947-s2.pdf]

Supplementary information

Horizontal sliding of kilometre-scale hot spring area during the 2016 Kumamoto earthquake

Takeshi Tsuji [1, 2*], Jun'ichiro Ishibashi [3], Kazuya Ishitsuka [4], Ryuichi Kamata [5]

1. International Institute for Carbon-Neutral Energy Research (I2CNER), Kyushu University, Japan
2. Department of Earth Resources Engineering, Kyushu University, Japan
3. Faculty of Science, Kyushu University, Japan
4. Fukada Geological Institute, Japan
5. Chiiki-Shigen-Kaihatsu, Japan

* Correspondence and requests for materials should be addressed to T. Tsuji (tsuji@i2cner.kyushu-u.ac.jp)

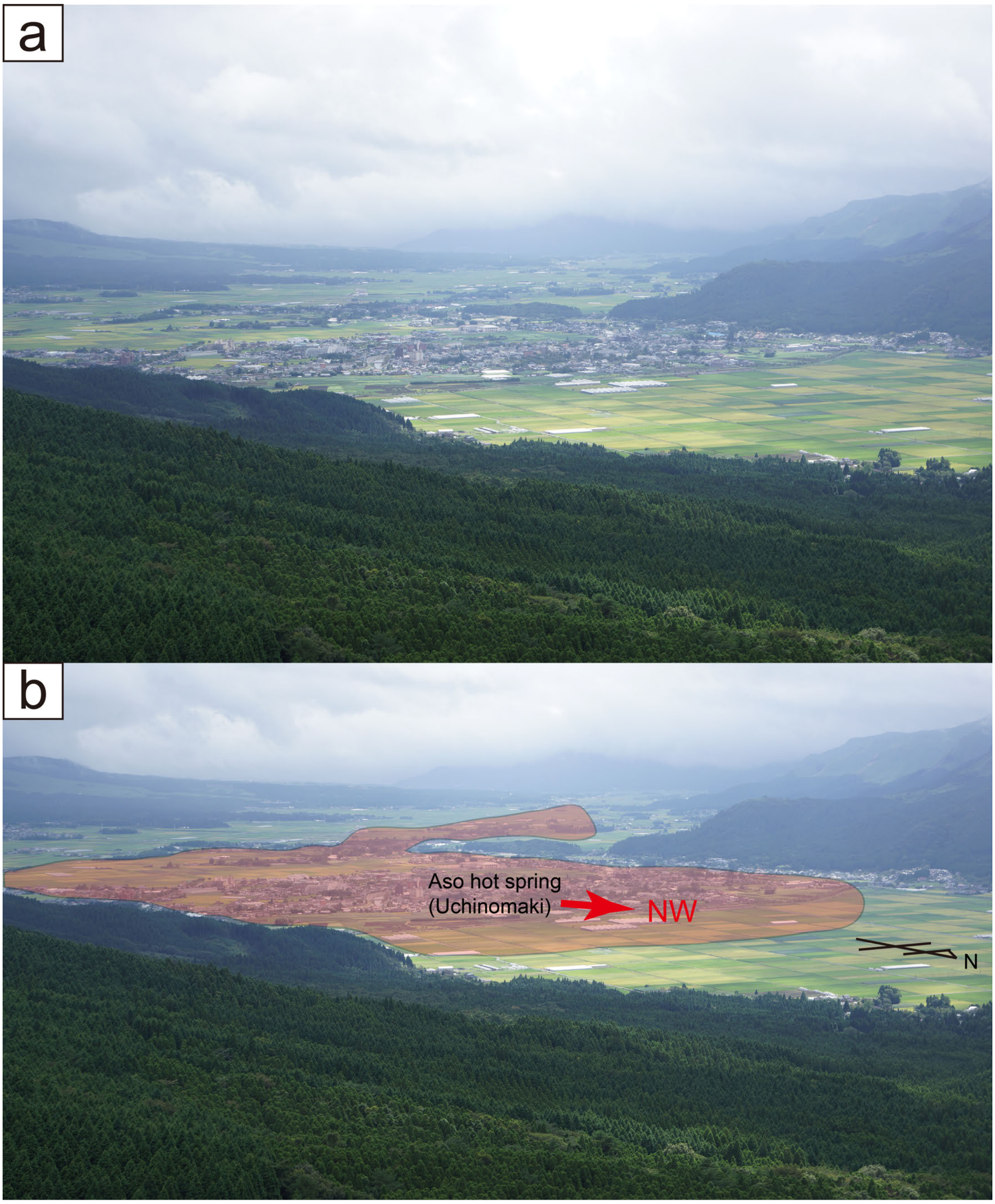

Supplementary Figure S1. Picture of the Aso hot spring area. In panel (b), we highlight the area that was horizontally displaced in the NW direction during the main shock (red shaded area).

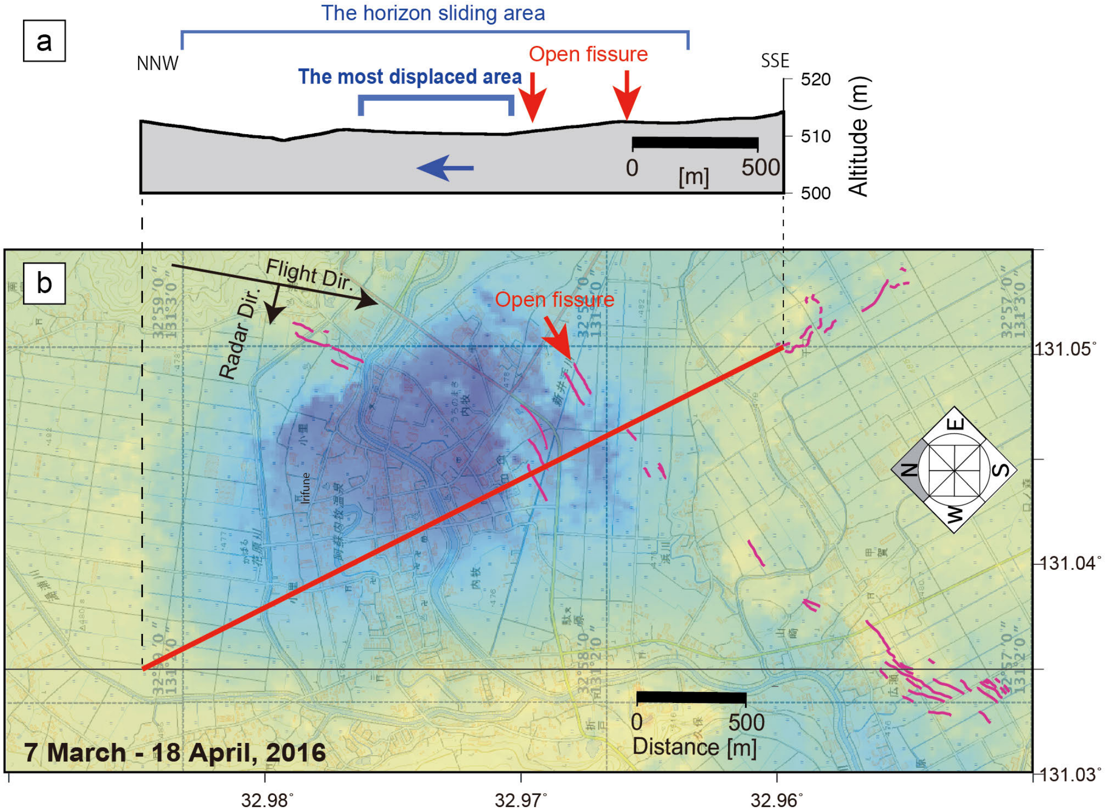

Supplementary Figure S2. Surface altitude around the Aso hot spring (Uchinomaki area). The location of cross-section in panel (a) is red line in panel (b). The cross-section in panel (a) is for sliding (NW) direction of geologic block. The surface deformation around the hot spring (panel b) is same as Fig. 2c. The regional map in panel (b) published by the Geospatial Information Authority of Japan (http://www.gsi.go.jp/).

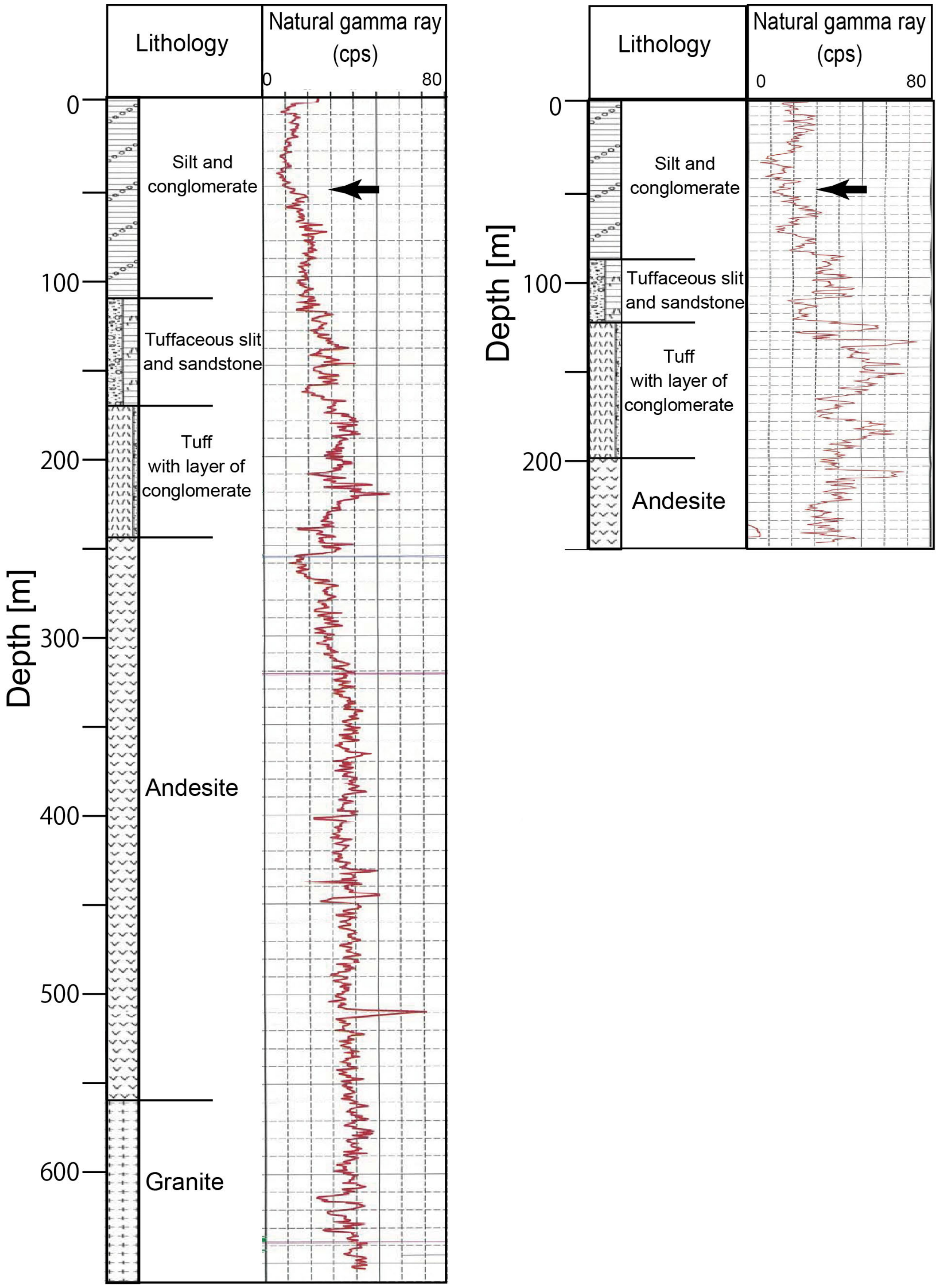

Supplementary Figure S3. Lithology and logging data (natural gamma ray) at the Aso hot spring area. The logging data of left panel was obtained at well A, and the data of right panel was obtained at ~200 m south of well A. The black arrows indicate the sliding formation.

Supplementary Video S1. **Video of the borehole (well B) from 49.5 to 51 m via borehole camera.** The location of well B is displayed in Fig. 2c. The borehole (casing pipe) is largely tilted deeper than 50 m. The pump displayed in this movie (at ~51.5 m) was collapsed due to bending of the borehole.
